# Prevalence of CYP17A1 gene mutations in 17α-hydroxylase deficiency in the Chinese Han population

**DOI:** 10.1186/s40885-019-0128-6

**Published:** 2019-10-15

**Authors:** Menglin Wang, Hao Wang, Haiying Zhao, Ling Li, Min Liu, Fujia Liu, Fansen Meng, Caini Fan

**Affiliations:** 1 Department of Hypertension, Henan Provincial People’s Hospital; People’s Hospital of Zhengzhou University, Zhengzhou, 450003 Henan China; 2 Bestnovo Beijing Medical Laboratory, No.14 Building, Room 502, PKUCare Industry Park, Zhong Guan Cun Life Science Park, Chang Ping District, Beijing, China

**Keywords:** 17α-hydroxylase deficiency, Congenital adrenal hyperplasia, Hypertension, CYP17A1, Gene mutation

## Abstract

**Background:**

17α-hydroxylase deficiency is a rare autosomal recessive disorder caused by mutations in the cytochrome P450 family 17 subfamily A member 1 gene. The major clinical presentation includes hypertension, hypokalemia, male pseudohermaphroditism and female gonadal dysplasia. Hundreds of pathogenic variants have been reported in this disorder, and some common mutations were found to be race-specific.

**Case presentation:**

In this study, we reported 5 Chinese girls with 17α-hydroxylase deficiency from Henan Province. The patients all came to the hospital for hypertension, and they also presented with sexual infantilism. The average age of the patients was 14 years old, ranging from 12 to 17 years old. They all had reduced blood cortisol, estradiol (E2), and testosterone (TESTO) and increased adrenocorticotropic hormone (ACTH), follicle-stimulating hormone (FSH), and luteinizing hormone (LH). They all had the appearance of females; however, three of the chromosome karyotypes were 46XX, and two were 46XY.

**Conclusions:**

All of the patients carried a mutation on the 329 amino acid of CYP17A1 exon 6. By summarizing the currently known pathogenic mutations of 17α-hydroxylase deficiency, we demonstrated the prevalence of these gene mutations in Chinese Han and non-Chinese populations.

**Electronic supplementary material:**

The online version of this article (10.1186/s40885-019-0128-6) contains supplementary material, which is available to authorized users.

## Background

Congenital adrenal hyperplasia (CAH) is a genetic disease caused by a deficiency of steroid hormone synthesis enzyme. CAH includes a group of autosomal recessive disease subtypes involving 21-hydroxylase deficiency [1], 11β-hydroxylase deficiency [2], 3β-hydroxysteroid hydrogenase deficiency [3], 17α-hydroxylase deficiency (17α-OHD) [4] and congenital lipoid adrenal hyperplasia (CLAH) [5]. 17α-OHD is a rare type of CAH and accounts for 1% of CAH [6, 7]. 17α-OHD is caused by a mutation in the cytochrome P450 family 17 subfamily A member 1 (CYP17A1) gene, which encodes 17α-hydroxylase, which leads to an imbalance of adrenal cortex and sex gland hormones and therefore low plasma cortisol and sexual hormone as well as high adrenocorticotropic hormone (ACTH). Clinically, 17α-OHD is characterized by hypertension, hypokalemia, male pseudohermaphroditism and female gonadal dysplasia. The diagnosis of 17α-OHD is based on a comprehensive overview of clinical, biochemical and molecular features. However, the clinical and biochemical presentations of this disorder are highly variable, and 10–15% of patients are normotensive at diagnosis [8]. Therefore, genetic diagnosis is crucial for diagnostic confirmation. Here, we reported five 17α-OHD teenagers presenting in our hospital within the past 4 years whose diagnoses were all confirmed via genetic testing. Surprisingly, genetic testing also revealed their molecular generality and hence implied similar molecular pathogenesis. We also summarized the 17α-OHD-causing mutations and described mutations common in the Chinese population, which demonstrated race-dependent differences.

## Case presentation

### The details of patient hospitalization

From October 2013 to December 2017, five female teenagers (social gender) with hypertension were hospitalized in the Hypertension Department of Henan Provincial People’s Hospital and received a final diagnosis of 17α-OHD. The average age of the patients was 14 years, with a range from 12 to 17 years old.

Patient 1, a 17-year-old female, was hospitalized because of a headache and giddy feelings lasting for 2 months and hypertension for 1 month. Her average daytime systolic blood pressure was 134 mmHg, her diastolic blood pressure was 96 mmHg, and her heart rate was 86 beats per minute. She had a normal BMI but had immature breasts and vulva and no menstruation, hirci or pubic hair. Hormone testing showed decreased plasma estradiol (E2), testosterone (TESTO) and renin activity but increased progesterone (PROG), follicle-stimulating hormone (FSH), luteinizing hormone (LH), aldosterone and aldosterone-renin ratio (ARR) (Table 1). She had reduced blood cortisol and increased ACTH. Further examination indicated that she had hypertension fundus changes, and her urine microalbuminuria test was more than 150 mg/l. Pelvic magnetic resonance imaging (MRI) did not detect the uterus or ovaries. Her chromosome karyotype was 46XY.

**Table 1:** Clinical characteristics of five 17α-hydroxylase deficiency cases. Serum cortisol, ACTH, FSH, LH, testosterone, estradiol and progesterone were measured using chemiluminescent immunoassays. Plasma renin activity and aldosterone were determined by the radio-immunity method

Clinical information	Patient 1	Patient 2	Patient 3	Patient 4	Patient 5	Normal value
Age (years old)	15	14	13	12	13	
Social Gender	Female	Female	Female	Female	Female	
Blood pressure (mmHg)	134/96	169/136	158/105	165/120	187/127	
Blood potassium (mmol/l)	4.29	3.52	3.32	2.96	2.7	3.5–5.5 mmol/l
Plasma renin activity(laying)	0.2	0.2	NA	0.2	0.2	0.15–2.33(ng.ml)/h
Plasma renin activity (standing)	0.2	0.2	0.2	0.2	0.2	0.1–6.56(ng/ml)/h
Aldosterone(laying)	79.9	103	NA	111	127	30-160 pg/ml
Aldosterone (standing)	68.1	108	81.4	133	158	70-300 pg/ml
ARR	34.05	54	40.7	66.5	79	
AngiotensinII (laying)	34.3	44.9	NA	39.6	87.2	30-160 pg/ml
AngiotensinII (standing)	50.9	51.9	50.2	51.6	96.3	70-300 pg/ml
Blood cortisol (0 am)	0	0.01	0.01	0.3	NA	0
Blood cortisol (8 am)	0.32	0.3	0.01	0.6	0.033	6.7–22.6μg/dl
Blood cortisol (4 pm)	0.04	0.19	0.11	0.4	0.015	3.35–11.3μg/dl
Blood ACTH (0 am)	16.2	12.4	13.7	41.1	NA	pg/ml
Blood ACTH (8 am)	90.1	84.5	33.6	373	168.5	12-46 pg/ml
Blood ACTH (4Pm)	16.2	12.4	13.7	41.1	86.9	6-23 pg/ml
E2	12.19	9.36	0.01	17.1	4.14	Children:male0–41 IU/L, female0–50 IU/L
PROG	11.4	14.25	7.43	13.46	10.55	0–1.2 IU/L
TESTO	0.25	0.24	0.1	0.17	0.01	Children:male0.45–2.26 IU/L, female0.03–1 IU/L
FSH	69.06	67.76	76.86	31.53	117.27	Children:male0-10 IU/L; femal0-15 IU/L
LH	26.42	29.81	26.25	10.14	59.38	Children:male0-12 IU/L, female0-15 IU/L
T3	5.68	5.87	5.49	7.22	NA	3.5–6.5 pmol/l
T4	15.6	17.53	16.46	19.44	NA	11.5–22.7 pmol/l
TSH	1.846	1.946	3.447	1.033	NA	0.55–4.78uIU/ml
Urine microalbuminuria(mg/l)	> 150	12.87	439.19	NA	NA	< 150 mg/l
Urine protein	+	–	NA	+	NA	
Urine protein(24 h)	0.1	NA	NA	NA	NA	0–1.5 g/24 h
Hypertension Fundus changes	Positive	Negative	Positive	Negative	NA	
Chromosome karyotype	46XY	46XX	46XX	46XY	46XX	
SRY	Positive	Negative	Negative	Positive	Negative	
Pelvic MRI	No uterus and ovaries	uterus and ovaries	uterus and ovaries	No uterus and ovaries	uterus and ovaries	
mutation	c.985_987delinsAA (p.Y329Kfs) c.1459_1467 (p.487_489del)	c.987delC p.Y329	c.985_987delinsAA (p.Y329Kfs)	c.985_987delinsAA (p.Y329Kfs)	c.985_987delinsAA (p.Y329Kfs)	
homozygote	heterozygote	homozygote	homozygote	homozygote	heterozygote	

*NA* not available, *E2* estradiol, *TESTO* testosterone, *PROG* Progesteron

Patient 2 was a 14-year-old girl who was hospitalized because of hypertension lasting for 2 months. Her hypertension was also found in a school physical examination. Her average daytime blood pressure was 169/136 mmHg, and her heart rate was 83 bpm. She had normal BMI but immature breasts and vulva and had not experienced menstruation. Blood potassium was 3.52 mmol/l, which was on the low threshold. The hormone test indicated a disorder with decreased plasma E2, TESTO and renin activity but increased PROG, FSH, LH, aldosterone and ARR. She had reduced blood cortisol and increased ACTH (Table 1). Pelvic MRI showed an immature uterus and ovaries. Her chromosome karyotype was 46XX.

Patient 3 was a 13-year-old female who was hospitalized because of hypertension accompanied by headache lasting for 2 months. She was found to have high blood pressure at a school physical examination. Her average daytime blood pressure was 158/105 mmHg, and her heart rate was 85 bpm. Blood potassium was slightly lower than the normal value of 3.32 mmol/l. She had normal BMI but immature breasts and vulva and had not experienced menstruation. Her plasma E2, TESTO and renin activity were all decreased, whereas her PROG, FSH, LH, aldosterone and ARR were increased. Her blood cortisol was reduced, and ACTH was increased. She had hypertension fundus changes, and the result of the urine microalbuminuria test was 439.9 mg/l, which suggested that she had hypertension complications. Pelvic MRI displayed her immature uterus and ovaries. Her chromosome karyotype was 46XX.

Patient 4, who had the appearance of a 12-year-old girl, was hospitalized because she had felt dizzy for 1 week. Her average daytime blood pressure was 165/120 mmHg, and her heart rate was 90 bpm. Blood potassium was extremely low, with a value of 2.96 mmol/l. She had a normal BMI but immature breasts and vulva and had not experienced menstruation. Hormone analysis showed that she had hormone disorder with reduced plasma E2, TESTO and renin activity but enhanced PROG, FSH, LH, aldosterone and ARR (Table 1). She had reduced blood cortisol, and ACTH was extremely high. Pelvic MRI could not find her uterus and ovaries but showed suspected testis. Her chromosome karyotype was 46XY.

Patient 5 was a 13-year-old girl who was hospitalized because of high blood pressure lasting for 2 months. Her average daytime blood pressure was 187/127 mmHg, and her heart rate was 76 bpm. Blood potassium was extremely low, with a value of 2.7 mmol/l. She had a normal BMI but immature breasts and vulva and had not experienced menstruation. Her plasma E2, TESTO and renin activity were all decreased, whereas PROG, FSH, LH, aldosterone and ARR were increased (Table 1). She had reduced blood cortisol and increased ACTH. Pelvic MRI showed an immature uterus and ovaries, and her chromosome karyotype was 46XX.

### Patient follow-up after treatment

Patient 1 was indicated to be a male according to the karyotype, but testicles were not found by imaging. Hence, he received intraperitoneal exploration, and cryptorchidism was found in his abdomen. Since cryptorchidism could have a malignant transformation, he was advised to undergo cryptorchidectomy. However, he was still hesitant. His blood pressure was well controlled by a calcium antagonist, spironolactone and cortisone. The main clinical features and biochemical and hormonal findings are summarized in Table 1.

Patient 4 was also found to be male by karyotype. However, he decided to be a female and underwent cryptorchidectomy. Medications including cortisone, estradiol, calcium carbonate D3 and alfacalcidol were used to maintain normal development.

Patients 2, 3 and 5 were confirmed to be female and directly given exogenous estrogens, including estradiol and progesterone, to maintain proper development of their genitals. At the same time, calcium antagonists, angiotensin II receptor blockers, spironolactone, calcium carbonate D3 and cortisone were used to control blood pressure. During follow-up, their blood pressure had decreased to normal, and the immature uterus and breasts had started to develop. Patient 5 also had normal menstruation (Additional file 1).

### CYP17A1 gene sequence analysis

**Fig. 1:**
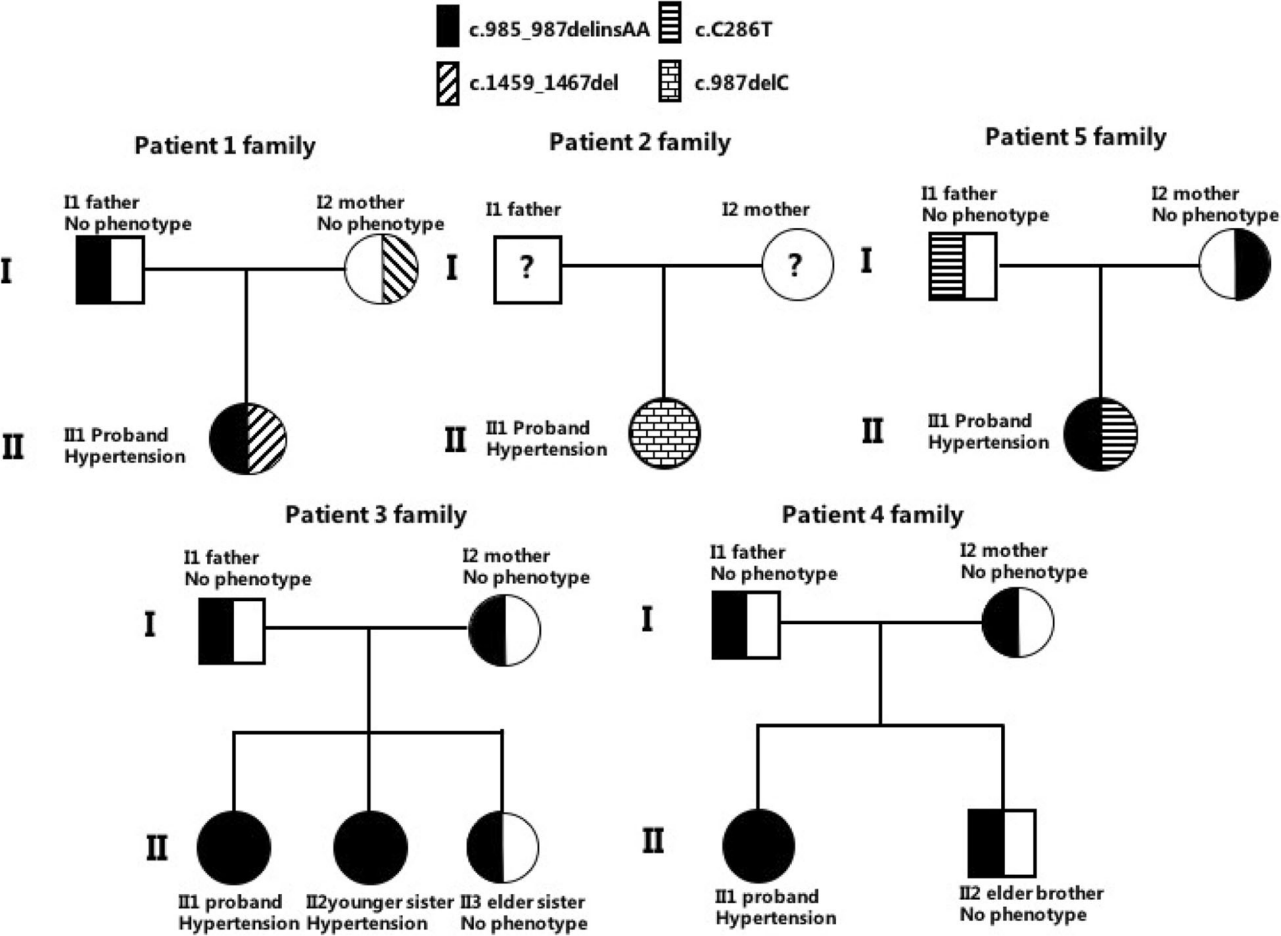
The pedigree of five patients. The family members were plotted according to the genotype and phenotype, and the detailed mutations were according to the legend

The entire coding region of the patient’s CYP17A1 gene, including the exon-intron boundaries, was sequenced. Pathogenic mutations were found in the CYP17A1 gene in all five patients. Patient 1 carried two variants of the CYP17A1 gene: the nonsense mutation c.985_987delinsAA (p. Y329Kfs) het and missing mutation c.1459_1467 (p.487_489del) het. Both CYP17A1 c.985_987delinsAA and c.1459_1467 (p.487_489del) had no frequency record in the 1000 Genomes Project (1000G) and ESP6500 databases, whereas they showed a very low frequency (1.291 × 10^− 5^ and 4.947 × 10^− 5^) in Exome Aggregation Consortium (ExAC), and they were confirmed to be class A mutations according to the American College of Medical Genetics (ACMG) standard. These two mutations were verified in her parents. Her father carried c.1459_1467 (p.487_489del) het, and her mother had c.985_987delinsAA (p. Y329Kfs) het (Fig. 1). Interestingly, we found that the other four patients also carried the mutation in the 985–987 region. Patient 5 was heterozygous, and patients 3 and 4 were homozygous for c.985_987delinsAA(p.Y329Kfs), whereas patient 2 was homozygous for c.987delC p.Y329*. The detailed pedigrees of these five families are shown in Fig. 1 and Table 1.

### 3D protein conformation

We mimicked the three-dimensional structure of human 17α-hydroxylase protein before and after *CYP17A1* mutation. PROCHECK software was used to evaluate the model’s reliability. It was reliable, with 83.4% amino acid residues in most favored regions, and 16% in additional allowed regions (Additional file 2: Figures S1&S2).

**Fig. 2:**
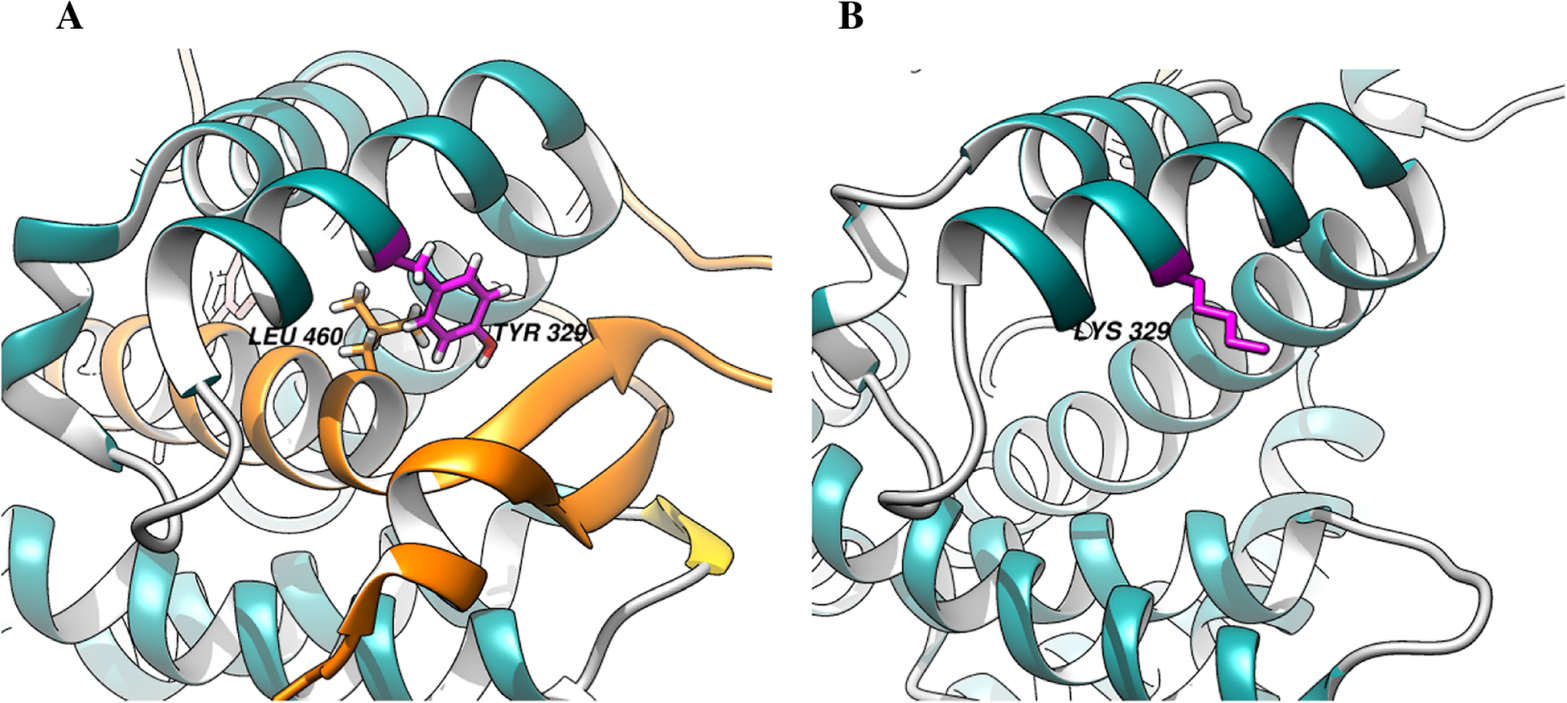
The CYP17A1 protein active center before (**a**) and after (**b**) amino acid changes. The CYP17A1 protein 3D model was mimicked by a computer. **a** Normal protein active center of 3D structure. The Van der Waals force between Tyr329 and Leu460 stabilized the protein active center. The region highlighted in orange was the missing part after mutation. **b** Mutated protein active center of 3D structure. The replacement of Tyr329 by Lys329 caused the loss of protein active center stability

According to the three-dimensional protein model, tyrosine 329 (Tyr329) was located in the center of the J-helix of 17α-hydroxylase and formed a hydrophobic portion interacting with the C terminal L460 of the L helix to stabilize the enzymatic structure (Fig. 2a). However, the replacement of Tyr329 by lysine (Lys) led to the loss of 418–503 amino acids, which directly damaged the enzyme active center (Fig. 2b).

**Fig. 3:**
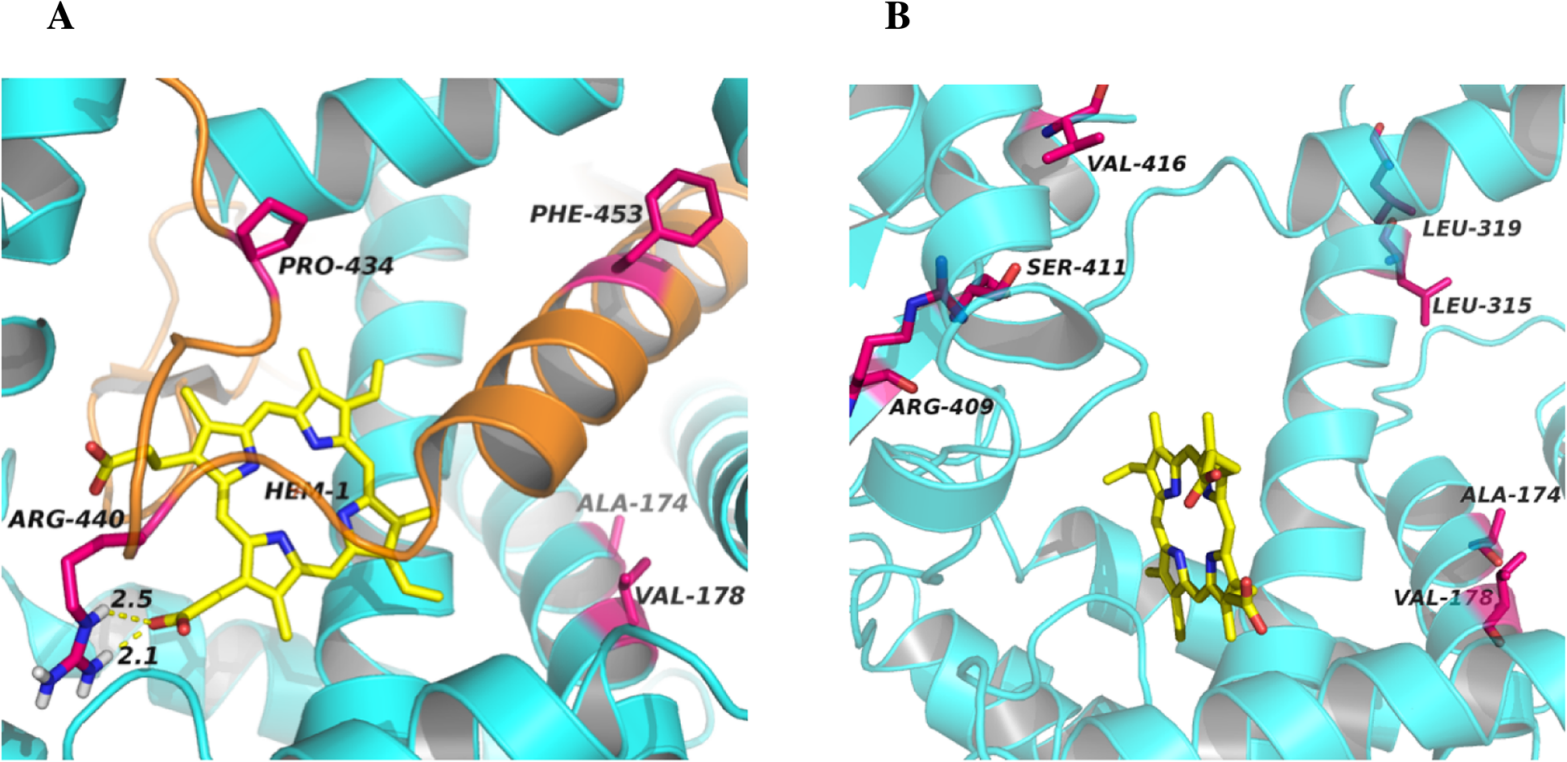
Ferroheme binding region before and after amino acid changes. **a** Normal ferroheme binding region in the protein active center. Arg440 and heme formed 2 hydrogen bonds in the protein active center, and Pro434 and Phe453 were essential in stabilizing the β-fold and J-helix structure around the active center. **b** Ferroheme binding region in the protein active center after mutation. The Tyr329Lys mutation caused a shortened protein missing the amino acids after 417, which directly damaged the functional region after the ferroheme binding region shifted after losing the hydrogen bond and the upper left shift of preserved Arg409, Ser411 and Val416

Furthermore, we know that the heme binding site (435-455aa) is a pivotal functional region of 17α-hydroxylase, in which Arg440 and heme formed 2 hydrogen bonds and Pro434 and Phe453 were essential in stabilizing the β-fold and J-helix structure around the active center, respectively (Fig. 3a). Nevertheless, the Tyr329Lys mutation caused a shortened protein missing the amino acids after 417, which brought about the loss of the heme binding region (435-455aa) and therefore 17α-hydroxylase functional deficiency. (Fig. 3b).

## Discussion and conclusions

The first 17α-OHD case was reported in 1966 by Biglieri et al. [9], and it involved a 35-year-old female with the initial symptom of hypertension, who was short in stature and had delayed menses. Subsequently, New et al described a male 17α-OHD case characterized by male pseudohermaphroditism, ambiguous external genitalia, and the absence of male secondary sexual characteristics in 1970 [10]. The first Chinese case was described by Shanghai Rui Jin Hospital in 1982 [11]. To date, more than 500 cases have been reported.

**Fig. 4:**
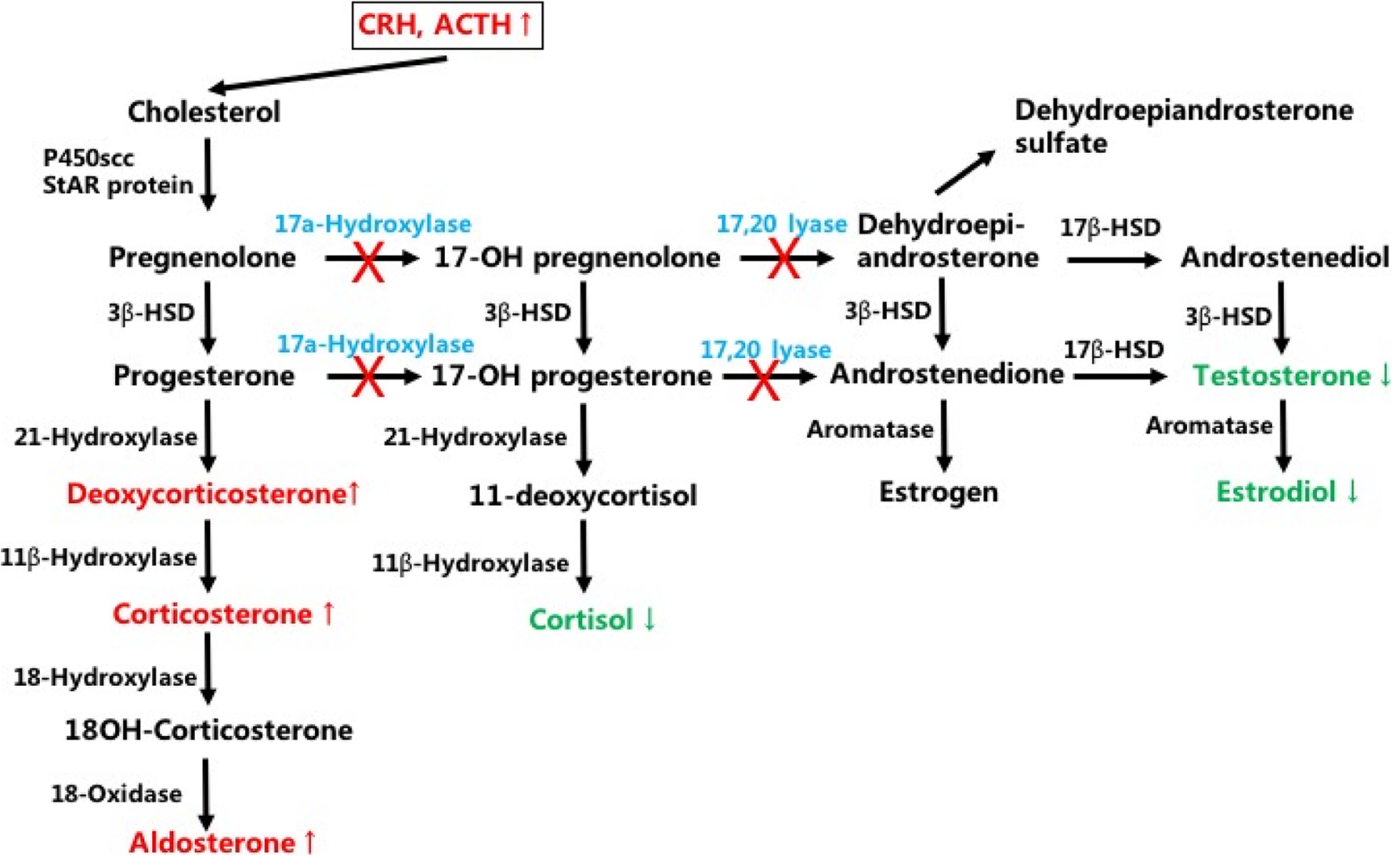
The hormone synthetizing way of the adrenal gland and gonads regulating by 17α-hydroxylase and 17,20-lyase

Loss of function of 17α-hydroxylase caused by the CYP17A1 mutation could explain the whole 17α-OHD pathogenesis. 17α-hydroxylase is the key enzyme that regulates hormone synthesis in the adrenal gland and gonads. It is composed of 504 amino acids and is a mixed functional enzyme with the combined activity of both 17α-hydroxylase and 17, 20-lyase [6] (Fig. 4). 17α-hydroxylase is mainly located in the testis Leydig cells, follicular ovarian cells, and the zona fasciculata and zona reticularis of the adrenal grand but not the zona glomerulosa. Catalyzed by 17α-hydroxylase, pregnenolone and progesterone are transformed into 17-OH pregnenolone and progesterone, respectively, which are further cleaved by 17,20-lyase to form the precursors of estrogen, dehydroepiandrosterone (DHEA) and androstenedione (ASD) [12]. In the adrenal gland, 21- and 17α-hydroxylase hydroxylates progesterone to produce deoxycorticosterone (DOC) and 11-deoxycortisol and finally mineralocorticoid and hydrocortisone, respectively. Conversely, deficiency of 17α-hydroxylase causes the accumulation of corticosterone and aldosterone, which brings about hypertension and hypopotassemia. At the same time, deficiency of 17α-hydroxylase also leads to cortisol and sexual hormone disorder and, consequently, the symptoms of male pseudohermaphroditism and female gonadal dysplasia. As feedback to the reduced cortisol synthesis, the pituitary could excrete excess ACTH, which may lead to bilateral adrenocorticohyperplasia.

17α-hydroxylase is encoded by the CYP17A1 gene, which was first cloned in 1987 [13]. It is located on chromosome 10q24–25 and is composed of 8 exons and 7 introns, with a total length of 5.7 kb [14, 15]. To date, 119 mutations in exons and introns have been considered pathogenic or disease-causing mutations (DM) in 17α-OHD according to the ClinVar and HGMD databases, including missense mutations, insertions, deletions and duplications, and frameshift (fs) mutations (Additional file 2: Table S1). Among the five patients we reported here, patients one and five carried compound heterozygous mutations, whereas all the others had homozygous mutations. Surprisingly, all five patients carried the mutation encoding the same protein position tyrosine-329 with c.987delC in patient 3 and c.985_987delinsAA in other patients. The nucleotide 985 to 987 (TAC) of CYP17A1 was replaced by AA in exon 6 via 985 T > C and 987delC to form a deletion/insertion mutation, causing a frameshift with tyrosine (Y)-329-Lysine(K) as the first affected amino acid. This event finally led to the premature stop codon of 418TGA and the formation of a shortened protein with 417 amino acids without the pivotal functional domain – the heme-binding region (435-455aa). The c.985_987delinsAA (p.Y329K) mutation in CYP17A1 causes complete loss of 17α-hydroxylase activity in vivo and produces low-density metabolic products in vitro [16].

**Fig. 5:**
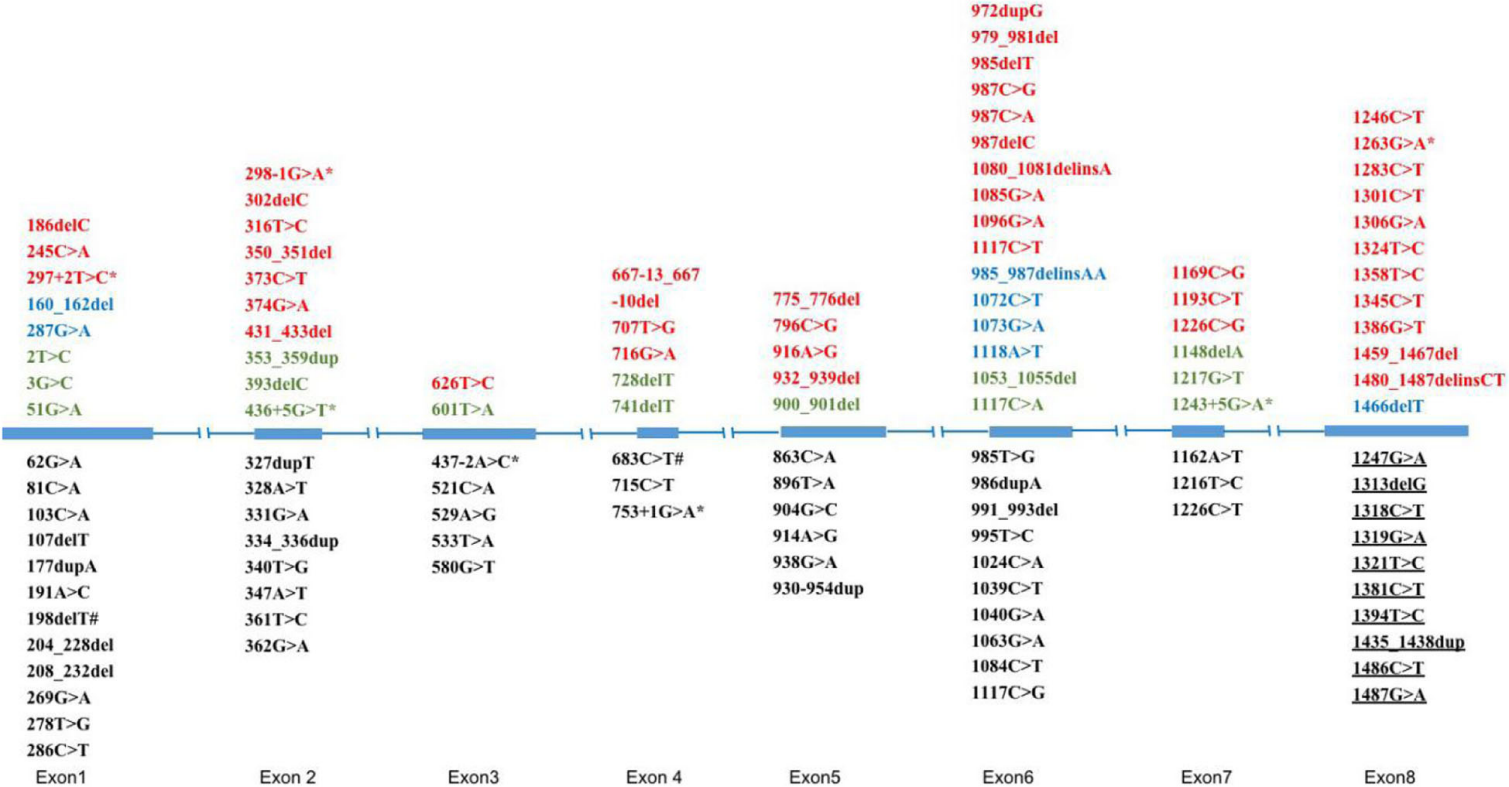
The 17α-OHD mutations in Chinese, Asian and other races. Red indicates Chinese; green indicates Asian; blue denotes mutations in both Chinese and Asian populations; Black indicates non-Chinese and non-Asian populations. Note: # means DM?; *indicates splicing mutation; del, deletions; dup, insertions/duplication; delins, indels; the following mutations were carried but not mapped in the figure due to restricted space: gross deletions: exons 1–6, c.(?_–345)_1039 +?del; exons 1–6, c.(?_-172)_(1139 + 1_1140–1)del. Complex rearrangements: exons 1–6, del. c.-2011_436 + 119 and del c.437-93_1140–262; exons 2–3, del. 518 bp affecting parts of ex. 2–3, ins. 469 bp

This mutation was initially reported by a Korean group as a compound heterozygote in a Korean individual with primary amenorrhea, hypokalemic, hypertension and karyotype 46 XX [17] in 2003. This mutation was widely detected in Chinese 17α-OHD patients [18, 19]. According to their observation, Tian et al. assumed that mutations of 17α-OHD varied according to race and that the c.985_987delinsAA mutation was common in Asian people [20]. A study of 15 Chinese 17α-OHD patients reported that 10 out of 15 (66.7%) patients carried the c.985_987delinsAA (p.Y329K) mutation, with two homozygotes and 8 heterozygotes [21]. To explain the high prevalence of the c.985_987delinsAA (p. Y329K) mutation, a possible founder effect of this mutation has been described [22]. However, the prevalence of 17α-OHD mutations in China remains elusive. Han et al indicated that approximately 90% of 17α-OHD patients carried at least one of the mutations (c.985_987delinsAA (p.Y329K); c.1459_1467 (p.487_489del)) [16]. To clarify this, we searched all published 17α-OHD English papers from PubMed and summarized the CYP17A1 gene mutations of all reported cases. A total of 133 English papers introduced 17α-OHD cases, and 111 of these studies performed genetic testing. In total, genetic mutations were reported in 181 Chinese cases, of which 70 (38.6%) had c.985_987delinsAA (p.Y329K), 55 (30.4%) had c.1459_1467del (p.487_489del) mutations, and the remaining 43 (31%) had other mutations. In contrast, 92.7% of cases (231 out of 249 cases) carried mutations other than c.985_987delinsAA (p.Y329K) and c.1459_1467 (p.487_489del) in non-Chinese individuals. Only 18 cases carried 329 amino acid mutations: 8 Korean (3.2%) patients out of 249 non-Chinese patients carried the c.985_987delinsAA (p.Y329K), 1 Japanese (0.4%) patient carried Y329X iA [23], 3 Brazilian (1.2%) patients had Y329D [24], and 6 Brazilian patients had Y329X [7]. The prevalence of Chinese 17α-OHD mutations is shown in Fig. 5.

To further understand how the gene mutation influenced the protein 3D conformation, we mimicked the protein 3D structure of 17α-hydroxylase by computer. The 3D conformation of 17α-hydroxylase has been reported in a previous study, which showed that the replacement of tyrosine 329 with a charged aspartate weakened the hydrophobic interaction and destabilized the enzymatic structure but did not directly perturb the active site [7]. Here, we first created a 17α-hydroxylase protein 3D model and mimicked the replacement of tyrosine 329 with lysine. The heme binding site (435-455aa) is a pivotal functional region of 17α-hydroxylase. Arg440 and heme formed 2 hydrogen bonds, and Pro434 and Phe453 stabilized the β-fold and J-helix structures around the active center, respectively (Fig. 3a). The replacement of Tyr329 by lysine (Lys) caused the loss of 418–453 amino acids, directly damaging the heme binding site, the enzymatic active center (Fig. 3b). Hence, the c.985_987delinsAA mutation was powerful enough to damage the functional heme region of 17α-hydroxylase and consequently led to the loss or reduction of 17α-hydroxylase activity.

In our study, all patients demonstrated clinical characteristics of 17α-OHD, including hypertension and secondary sexual disorder. Hypokalemia was found in four patients, and one patient had normal blood potassium. All five cases were teenagers who had exhibited hypertension symptoms approximately 8 weeks before hospitalization. However, patients 1 and 3 already had urine microalbuminuria and decreased circadian variation in blood pressure; specifically, patient 1’s blood pressure changed to the reverse spoon shape, implying that their blood pressure could be abnormal far beyond the duration of observed symptoms. Compared to adults, the teenagers were too young and experienced more severe hypertension-related complications. Hence, the early identification and diagnosis of young patients could contribute to well-controlled blood pressure and reduce subsequent disease-related complications.

All five cases presented as females when hospitalized, but patients 1 and 4 were identified as males with 46XY after karyotype examination. It was hard for these patients to accept the fact that they were actually not girls, and they had to decide their gender since it would influence ongoing therapy. Patient 1 was severely troubled by gender selection and determined to delay cryptorchidectomy. However, he could be in danger of malignant transformation of testosterone by postponing the surgery. Early identification and diagnoses of 17α-OHD patients could resolve problems with gender selection earlier. Due to the variable clinical and biochemical presentations of this disorder, genetic tests, especially high frequency variant screening for c.985_987delinsAA (p.Y329K) and c.1459_1467 (p.487_489del), for example, could be helpful for the early identification of 17α-OHD patients.

In summary, we reported five patients with 17α-OHD, first mimicked the 3D protein model of 17α-hydroxylase after c.985_987delinsAA (p.Y329K) mutation and showed the damaged enzymatic active center after mutation. We first systematically summarized the currently known pathogenic mutations of 17α-OHD and demonstrated their prevalence in Chinese Han and non-Chinese populations. We believe that genetic testing is of great clinical significance for these young patients with hypertension.

## Additional files

Additional file 1: Shows materials and methods (DOCX 13 kb)

Additional file 2: 17a hydroxylase deficiency related mutations, and CYP17A1 protein three dimension structure pull graph in more detail (DOCX 299 kb)

## Data Availability

We have put our supporting data in additional files.

## Abbreviations

17α-OHD: 17α-hydroxylase deficiency
ARR: Aldosterone renin ratio
ASD: Androstenedione
BMI: Body mass index
CAH: Congenital adrenal hyperplasia
CYP17A1: Cytochrome P450 family 17 subfamily A member 1
DHEA: Dehydroepiandrosterone
DOC: Deoxycorticosterone
E2: Estradiol
FSH: Follicle-stimulating hormone
LH: Luteinizing hormone
MRI: Magnetic resonance imaging
PCR: Polymerase chain reaction
PROG: Progesterone
TESTO: Testosterone
