# Supplementary material for: Prevalence of CYP17A1 gene mutations in 17α-hydroxylase deficiency in the Chinese Han population
Source: Clin Hypertens. 2019 Oct 15;25:23. doi: 10.1186/s40885-019-0128-6 (PMC6792268; doi:10.1186/s40885-019-0128-6)
Supplement: Supplementary file 1 — Shows materials and methods (DOCX 13 kb) [file 40885_2019_128_MOESM1_ESM.docx]

**Materials and Methods**

Hormonal measurements

Serum cortisol, ACTH, FSH, LH, testosterone, estradiol, progesterone, plasma renin activity and plasma aldosterone concentration were measured using chemiluminescent immunoassays.

DNA isolation and next generation sequencing

All of the libraries were prepared based on the protocols of Agilent SureSelect QXT Library Prep Kit (5500-0127). Exome capture was prepared based on the protocols of Agilent SureSelect QXT Target Enrichment for Illumina Multiplexed Sequencing version E0. Before hybridization, library DNA quantity and quality were assessed by Agilent 2200 TapeStation. The DNA libraries were mixed with capture probes of targeted regions using the SureSelect Human All Exon V6 kit. The hybridization was performed at 65 °C for 1 hour to ensure targeted regions bind to the capture probes thoroughly. Streptavidin beads were used to capture probes containing the targeted regions of interest. Three wash steps with different wash buffer were done to remove non-specific binding from the beads.

The enriched libraries on the beads were then amplified by polymerase chain reaction (PCR). The PCR products were cleaned with Ampure XP beads (Agencourt, Boston, MA, USA) according to the SureSelect QXT protocol and then validated by Agilent 2200 TapeStation and qPCR for quality control analysis. The libraries were denatured and diluted to optimal concentration and applied in the cluster generation steps. Illumina NovaSeq 6000 S2 Reagent Kit (300 cycles) was used for paired-end 2×150 bp sequencing on an Illumina NovaSeq 6000 System.

Computer modelling of human 17α-hydroxylase

The crystal structure of human CYP17A1 (PDBID 5IRQ) with a resolution of 2.2 Å was chosen as the reference for the three-dimensional (3D) model of human cytochrome P45017A1 (17α-hydroxylase). To further understand how the mutation of CYP17A1 gene affect 17α-hydroxylase activity, we predicted the mutational amino acid sequence, such as insertion and deletion, using a database-search approach included in the software package ExASy (https://web.expasy.org/translate/). We also constructed a three-dimensional computer model using PHYRE software (version 2.0; http://www.sbg.bio.ic.ac.uk/phyre2/). According to the alignment obtained by the fold recognition procedure, amino acid residues were changed in the template. These model structures were energy-minimized using the steepest descent algorithm implemented in the SYBYL program package. PROCHECK (version 3.0; http://servicesn.mbi.ucla.edu/PROCHECK/) was used to assess the stereochemistry, the fitness of sequence and structure, and the total protein potential for the selected P450c17 model. To inspect the binding conformation of ferroheme in mutational protein, we docked the molecular into the original active site of mutational 17α-hydroxylase using Autodock4 software. The structural representations were generated using UCSF Chimera software (http://www.cgl.ucsf.edu/chimera/) and PyMOL 2.2 (https://pymol.org/2/).
