# Supplementary material for: Prevalence of CYP17A1 gene mutations in 17α-hydroxylase deficiency in the Chinese Han population
Source: Clin Hypertens. 2019 Oct 15;25:23. doi: 10.1186/s40885-019-0128-6 (PMC6792268; doi:10.1186/s40885-019-0128-6)
Supplement: Supplementary file 2 — 17a hydroxylase deficiency related mutations, and CYP17A1 protein three dimension structure pull graph in more detail (DOCX 299 kb) [file 40885_2019_128_MOESM2_ESM.docx]

**Supplementary Table. 1: 17a hydroxylase deficiency** **related mutations. The *CYP17A1* sequence refers to** NM_000102.3 sequence.

| **No** | **Chromosome Location** | **Protein** | **Clinical significance** | **Review status** |
| --- | --- | --- | --- | --- |
| 1 | CYP17A1, IVS3AS, 4-BP DEL |  | Pathogenic | NACP |
| 2 | CYP17A1, IVS2AS, A-C, -2 |  | Pathogenic | NACP |
| 3 | CYP17A1, 9-BP DEL |  | Pathogenic | NACP |
| 4 | CYP17A1, IVS7+5G-A |  | Pathogenic | NACP |
| 5 | CYP17A1, 1-BP DEL |  | Pathogenic | NACP |
| 6 | CYP17A1, 469-BP INS, 518-BP DEL |  | Pathogenic | NACP |
| 7 | CYP17A1, 7-BP DUP, EX2 |  | Pathogenic | NACP |
| 8 | c.1435_1438dupATCC | p.Pro480Hisfs | Pathogenic/Likely pathogenic | CP MS |
| 9 | c.1358T>C | p.Phe453Ser | Pathogenic | NACP |
| 10 | c.1283C>T | p.Pro428Leu | Pathogenic | NACP |
| 11 | c.1247G>A | p.Arg416His | Pathogenic | NACP |
| 12 | c.1216T>C | p.Trp406Arg | Pathogenic | NACP |
| 13 | c.1162A>T | p.Lys388Ter | Pathogenic | NACP |
| 14 | c.1084C>T | p.Arg362Cys | Pathogenic | NACP |
| 15 | c.1073G>A | p.Arg358Gln | Pathogenic | NACP |
| 16 | c.1040G>A | p.Arg347His | Pathogenic | NACP |
| 17 | c.1039C>T | p.Arg347Cys | Pathogenic | NACP |
| 18 | c.1024C>A | p.Pro342Thr | Pathogenic | NACP |
| 19 | c.985T>G | p.Tyr329Asp | Pathogenic | NACP |
| 20 | c.715C>T | p.Arg239Ter | Pathogenic | CPSS |
| 21 | c.601T>A | p.Tyr201Asn | Pathogenic | NACP |
| 22 | c.436+5G>T |  | Pathogenic | NACP |
| 23 | c.374G>A | p.Arg125Gln | Pathogenic | NACP |
| 24 | c.347A>T | p.Asp116Val) | Pathogenic | NACP |
| 25 | c.340T>G | p.Phe114Val) | Pathogenic | NACP |
| 26 | c.316T>C | p.Ser106Pro) | Pathogenic | CPSS |
| 27 | c.287G>A | p.Arg96Gln) | Pathogenic | NACP |
| 28 | c.286C>T | p.Arg96Trp) | Pathogenic | CPMS |
| 29 | c.278T>G | p.Phe93Cys) | Pathogenic | NACP |
| 30 | c.206_230del25 | p.Gly69Alafs) | Pathogenic | NACP |
| 31 | c.157_159delTTC | p.Phe54del) | Pathogenic | NACP |
| 32 | c.81C>A | p.Tyr27Ter) | Pathogenic | NACP |
| 33 | c.51G>A | p.Trp17Ter) | Pathogenic | NACP |
| 34 | c.3G>C | p.Met1Ile | DM | NA |
| 35 | c.2T>C | p.Met1Thr | DM | NA |
| 36 | c.103C>A | p.Pro35Thr | DM | NA |
| 37 | c.191A>C | p.Ty64Ser | DM | NA |
| 38 | c.245C>A | p.Ala82Asp | DM | NA |
| 39 | c.269G>A | p.Gly90Asp | DM | NA |
| 40 | c.328A>T | p.Lys110Term | DM | NA |
| 41 | c.331G>A | p.Gl111Ser | DM | NA |
| 42 | c.361T>C | p.Trp121Arg | DM | NA |
| 43 | c.362G>A | P.Trp121Term | DM | NA |
| 44 | c.373C>T | P.Arg125Term | DM | NA |
| 45 | c.521C>A | P.Ala174Glu | DM | NA |
| 46 | c.529A>G | Asn177Asp | DM | NA |
| 47 | c.533T>A | P.Val178Asp | DM | NA |
| 48 | c.580G>T | P.Glu194Term | DM | NA |
| 49 | c.626T>C | P.Leu209Pro | DM | NA |
| 50 | c.683C>T | P.Thr228Ile | DM? | NA |
| 51 | c.707T>G | P.Val236Gly | DM | NA |
| 52 | c.716G>A | P.Arg239Gln | DM | NA |
| 53 | c.796C>G | P.Leu266Val | DM | NA |
| 54 | c.863C>A | P.Ser288Term | DM | NA |
| 55 | c.896T>A | P.Ile299Asn | DM | NA |
| 56 | c.904G>C | P.Ala302Pro | DM | NA |
| 57 | c.914A>G | P.Glu305Gly | DM | NA |
| 58 | c.916A>G | P.Thr306Ala | DM | NA |
| 59 | c.938G>A | P.Trp313Term | DM | NA |
| 60 | c.987C>A | P.Tyr329Term | DM | NA |
| 61 | c.987C>G | P.Tyr329Term | DM | NA |
| 62 | c.995T>C | P.Ile332Thr | DM | NA |
| 63 | c.1063G>A | P.Ala355Thr | DM | NA |
| 64 | c.1072C>T | P.Arg358Term | DM | NA |
| 65 | c.1085G>A | P.Arg362His | DM | NA |
| 66 | c.1096G>A | P.Val366Met | DM | NA |
| 67 | c.1117C>A | P.His373Asn | DM | NA |
| 68 | c.1169C>G | P.Thr390Arg | DM | NA |
| 69 | c.1193C>T | P.Ala398Val | DM | NA |
| 70 | c.1217G>T | P.Trp406Leu | DM | NA |
| 71 | c.1226C>G | P.Pro409Arg | DM | NA |
| 72 | c.1226C>T | P.Pro409Leu | DM | NA |
| 73 | c.1246C>T | P.Arg416Cys | DM | NA |
| 74 | c.1301C>T | P.Pro434Leu | DM | NA |
| 75 | c.1306G>A | P.Gly436Arg | DM | NA |
| 76 | c.1318C>T | P.Arg440Cys | DM | NA |
| 77 | c.1319G>A | P.Arg440His | DM | NA |
| 78 | c.1321T>C | P.Ser441Pro | DM | NA |
| 79 | c.1324T>C | P.Cys442Arg | DM | NA |
| 80 | c.1345C>T | P.Arg449Cys | DM | NA |
| 81 | c.1381C>T | P.Gln461Term | DM | NA |
| 82 | c.1386G>T | P.Arg462Ser | DM | NA |
| 83 | c.1394T>C | P.Leu465Pro | DM | NA |
| 84 | c.1486C>T | P.Arg496Cys | DM | NA |
| 85 | c.1487G>A | P.Arg496His | DM | NA |
| 86 | c.298-1G>A | NA | DM | NA |
| 87 | c.437-2A>C | NA | DM | NA |
| 88 | c.753+1G>A | NA | DM | NA |
| 89 | c.1263G>A | NA | DM | NA |
| 90 | c.1243+5G>A | NA | DM | NA |
| 91 | c.667-13_667-10delTTTT | NA | DM | NA |
| 92 | c.107delT | NA | DM | NA |
| 93 | c.160_162delTTC | NA | DM | NA |
| 94 | c.186delC | NA | DM | NA |
| 95 | c.198delT | NA | DM？ | NA |
| 96 | c.302delC | NA | DM | NA |
| 97 | c.350_351delCT | NA | DM | NA |
| 98 | c.393delC | NA | DM | NA |
| 99 | c.431_433delAGA | NA | DM | NA |
| 100 | c.728delT | NA | DM | NA |
| 101 | c.741delT | NA | DM | NA |
| 102 | c.775_776delAT | NA | DM | NA |
| 103 | c.900_901delTG | NA | DM | NA |
| 104 | c.932_939delTTAAATGG | NA | DM | NA |
| 105 | c.979_981delAAG | NA | DM | NA |
| 106 | c.987delC | NA | DM | NA |
| 107 | c.985delT | NA | DM | NA |
| 108 | c.991_993delGAG | NA | DM | NA |
| 109 | c.1053_1055delCCT | NA | DM | NA |
| 110 | c.1148delA | NA | DM | NA |
| 111 | c.1313delG | NA | DM | NA |
| 112 | c.1459_1467delGACTCTTTC | NA | DM | NA |
| 113 | c.1466delT | NA | DM | NA |
| 114 | c.177dupA | NA | DM | NA |
| 115 | c.327dupT | NA | DM | NA |
| 116 | c.334_336dupATC | NA | DM | NA |
| 117 | c.353_359dupGCGCACA | NA | DM | NA |
| 118 | c.972dupG | NA | DM | NA |
| 119 | c.986dupA | NA | DM | NA |

Note: NACP: no assertion criteria provided; CP MS: criteria provided, multiple submitters, no conflicts;

CP SS: criteria provided, single submitter; DM, Disease-causing mutation; NA, not available.


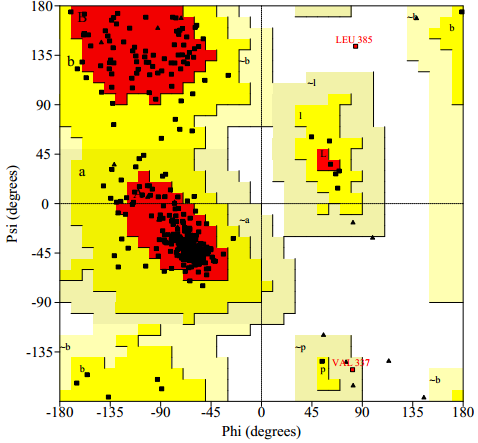


Supplementary Figure 1: CYP17A1 Protein three dimension structure pull graph. Red color indicated that the amino acid residues were in most favoured regions; yellow color suggested that amino acid residues were in additional allowed regions; light yellow implied that the amino acid residues were in generously allowed regions.


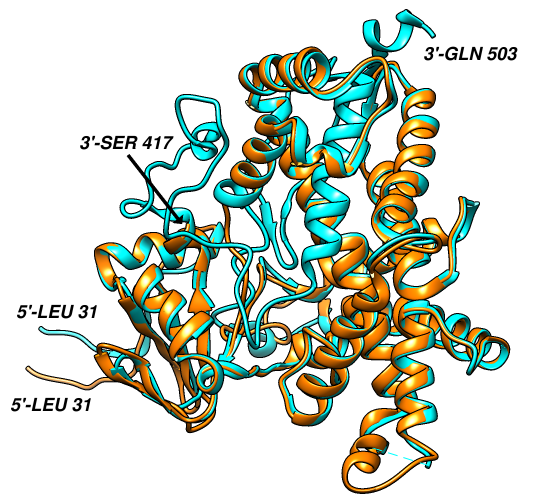


Supplementary Figure 2: the overlapping of CYP17A1 protein and its mutation protein. Blue color presented the normal CYP17A1 protein 3D structure; Orange color showed the 3D structure of mutation protein.
